# Medicinal Mushroom *Leucocalocybe mongolica* Imai Extracts Improve Mammary Gland Differentiation in Lactating Rats via Regulating Protein Expression

**DOI:** 10.1155/2022/5762847

**Published:** 2022-06-18

**Authors:** Asmaa Hussein Zaki, Bao Haiying, Li Zhijun

**Affiliations:** ^1^Key Laboratory of Edible Fungi Resources and Utilization, Ministry of Agriculture and Rural Affairs, Jilin Agricultural University, Changchun 130118, Jilin, China; ^2^College of Chinese Medicine Materials, Jilin Agricultural University, Changchun 130118, Jilin, China; ^3^Department of Agricultural Chemistry, Faculty of Agriculture, Minia University, Minya 61111, Egypt

## Abstract

*Leucocalocybe mongolica* is a known medicinal mushroom in China. It possesses many biological activities. This study investigated the effect of *L*. *mongolica* petroleum ether and water extracts (200, 500, and 1,000 mg/kg BW) on mammary gland differentiation during lactation. However, prolactin, growth hormone, progesterone, and estrogen levels were determined in serum by ELISA assay. Immunofluorescence, western blot, and real-time PCR were utilized to evaluate the expression levels of *β*-casein, *α*-Lactalbumin, prolactin receptor, progesterone receptor, and STAT-5a. The immunohistochemistry staining was used to detect the presence of steroid receptors. The results showed that petroleum ether and water extracts increased milk yield and milk content of calcium, total fat, total carbohydrate, and total protein. Prolactin and growth hormone levels were significantly upregulated in all treated groups compared with the control group. In contrast, progesterone and estrogen were downregulated. The high doses of petroleum ether and water extracts increased the expression levels of *β*-Cas, *α*-Lactalb, PRLR, PR, and STAT-5a. The observation of histological sections showed that the extracts induced higher mammary gland differentiation than the control group. This study is the first to use mushrooms as nutritional supplements to improve milk production and mammary gland differentiation during lactation.

## 1. Introduction

Breastfeeding is an essential source of nutrition for babies' growth and development. Breastfeeding protects infants from meningitis, bacterial infections, otitis media, tract infections, urinary tract infections, diarrhea, and respiratory disease [1–6]. Breast milk has also been associated with improved intelligence and cognitive development in children [7, 8]. According to WHO records, over 820,000 children die annually due to their inability to breastfeed or insufficient milk supply [9]. Although all mothers prefer breastfeeding during the suckling period, only a few mothers are able to continue breastfeeding for their babies [10]. The human mammary epithelial cell line MCF10 A is a popular in vitro model for researching normal breast cell activity and transformation. However, it is unclear if MCF10 A cells accurately resemble normal human mammary cells. MCF10 A cells were cultured in different culture types such as monolayer culture, suspension culture (mammosphere culture), three-dimensional (3D) “on-top” Matrigel, three-dimensional (3D) “cell-embedded” Matrigel, and combined Matrigel/collagen I gel cultures.

A wide range of hormones can regulate mammary gland milk production, including prolactin (PRL), growth hormone (GH), and progesterone (Pg) [11]. Prolactin is a primary hormone produced and released from the pituitary gland by lactotrophic cells [12]. On the other hand, the brain produces GH-releasing hormone, which promotes the pituitary gland secretion of growth hormone. Growth hormone plays a vital role in preserving lactation at the top level [13]. In contrast, it can be inhibited by somatostatin, which is likewise produced by the hypothalamus. Progesterone is primarily produced during the luteal phase of the menstrual cycle by the granulosa-lutein cells of the corpus luteum [14]. Progesterone has a significant physiological action in lobular-alveolar development in the mammary gland for milk secretion by inducing side branching of the ductal epithelium in the mammary glands [15].

Previous studies have provided many traditional herbal medicines used to increase milk production. *Leucocalocybe mongolica* is a medicinal and edible mushroom growing in Inner Mongolia's grassland, the north side of China, Russia's far east, and the north of Mongolia [16]. However, Mongolians consume it during pregnancy and lactation as a medicinal supplement to strengthen the mother's and baby's health and stimulate milk secretion [17]. Moreover, the fruiting body of *L*. *mongolica* is a vital biomedicine and source of a wide range of medically valuable chemicals that help the therapeutic potential for disease prevention and help maintain good health. The bioactive compounds in *L*. *mongolica*'s fruiting body are polysaccharides, proteins, peptides, phenolic compounds, triterpenoids, unsaturated fatty acids, flavonoids, alkaloids, and volatile components [18, 19]. Consuming antioxidant-rich foods such as flavonoids and phenolic compounds may help delay the aging of mammary alveolar cells [20]. On the other hand, ergosterol and laccase from *L*. *mongolica* demonstrated an inhibitory impact on MCF7 breast cancer cells [21, 22]. Overall, this study aims to evaluate petroleum ether and water extracts of *L*. *mongolica* to enhance milk yield and composition, mammary gland differentiation, and milk protein expression.

## 2. Materials and Method

### 2.1. Collection and Extraction of *Leucocalocybe mongolica*

Fruit body *L. mongolica* was obtained from the Xinqiao market of Hailar City, Inner Mongolia, China, and identified by Prof. Haiying Bao. The samples were dried in a 40°C oven for 7 days, crushed using a grinder, and dried in an airtight container. Mushroom samples of 500 g were mixed with 5 L petroleum ether for 8 hours in a Soxhlet instrument to extract the bioactive components. At the same time, 500 g of the mushroom powder was boiled with 5 L of water for 4 hours. The residues were removed after filtering, and the solvents from the supernatant were evaporated to dryness using a rotary evaporator under a vacuum. They were stirred in a water bath for 6 hours at 60°C to obtain powder extract and saved until use.

### 2.2. Cell Culture

MCF10 A cell line human mammary was obtained from Procell Life Science & Technology Co. Ltd, Wuhan, China. The cells were kept in RPMI 1640 medium with 10% FCS, 5 mg/mL insulin, 10 ng/mL EGF, 2 mM L-glutamine, 50 units/mL penicillin, and 50 mg/mL streptomycin (growth medium). The cells were cultured in a humidified incubator (5% CO_2_) at 37°C.

### 2.3. Cell Proliferation

Cell proliferation of the MCF10 A was evaluated using the Cell Counting Kit-8 assay (CCK-8). Briefly, cells were inoculated in a 96-well plate at a density of 5 × 10^5^ cells/well to attach for 24 h before being exposed to petroleum ether, water extracts, and metoclopramide (50, 100, and 200 *μ*g/mL) in a humidified incubator for 48 h (37°C, 5% CO_2_). After incubation, a CCK-8 reagent (10 *μ*L) was added to each well, followed by incubation for another 2 hour (37°C, 5% CO_2_). Finally, the absorbance was measured at 450 nm.

### 2.4. Animal

Forty-eight female Sprague–Dawley rats of three months old (bodyweight 200–220 g) were purchased from Changchun Yisi Experimental Animal Technology Co. Ltd. (China). The rats were divided randomly into eight groups (six mothers in each group and six pups/single mother). The eight lactating groups were divided into three groups: *L*. *mongolica* petroleum ether at high-dose HP (1,000 mg/kg bw/day), medium-dose MP (500 mg/kg bw/day), and low-dose LP (200 mg/kg bw/day) and three groups treated with *L*. *mongolica* water extract at high-dose HW (1,000 mg/kg bw/day), medium-dose MW (500 mg/kg bw/day), and low-dose LW (200 mg/kg bw/day). In addition, one group (MET) received metoclopramide at the dose of 5 mg/kg bw/day, and finally, the control (CON) group. Mothers started getting the doses 2 days postpartum. The extracts powder was dissolved in water (100 mg/mL) just before giving it to the rats according to their body weight (bw) and required dosage, while the control group took 1 mL water/day. All doses were taken orally using a stomach tube from 9:15 to 11:00 every morning. The animal center school approved the experimental procedures and animal care at Jilin Agricultural University in Changchun, China. Temperatures vary from 25 to 30°C with a 12 h light and 12 h dark cycle, and humidity ranges from 30 to 50%. All of the rats had unrestricted access to food and water for 20 days (the duration of the experiment). However, none of the pups died during the experiment time. The mothers were euthanized at 8:00 on the 21st day of lactation.

### 2.5. Milk Yield and Milk Sample Collection

The daily milk yield was measured by comparing the weights of rat pups before and after feeding. Every day, the pups were weighed at 8:00 to calculate the daily growth rate. After weighing at 9:00, they were separated from their dams and starved for 4 hours during the test period. At 1:00, they were weighed again. Then, they were returned to their dams, allowed to suck for 1 hour, and weighed again (final weight). Milk yield was measured as the mean weight increase in 1 hour for each litter as previously described in [23]. At the end of the 20th day of the experiment, the dams were separated from pups for about 20 hours (12:00 p.m. to next day 8:00 a.m.). During the dissection of mothers, milk samples (0.5–1 mL) were obtained freshly from the mammary gland tissue using a 3 mL syringe and kept at −80°C.

### 2.6. ELISA

Blood samples were obtained from the retro-orbital sinus of the eyes before euthanizing the mothers. Serum was separated from blood samples and kept at −80°C for hormone assays. The levels of prolactin (PRL), growth hormone (GH), progestin (Pg), and estradiol (E2) were measured using rat-specific ELISA kits (AndyGene Biotechnology Co., Beijing, China), as recommended by the kit manufacturers. Similarly, calcium, total protein, carbohydrate, and fat content were determined in milk samples according to the kits manufacturers (AndyGene Biotechnology Co., Beijing, China). Absorption was measured at 450 nm on an ELISA reader (Bio–Rad, Hercules, CA, USA).

### 2.7. Histopathological Analysis

The last pair of mammary gland tissues were isolated from each mother using sharp scissors after euthanasia and immediately fixed in 10% formaldehyde solution for 24 hours. After fixation, the sections were embedded in paraffin and cut into 5 µm slices; then hematoxylin and eosin staining was performed as directed by the manufacturers of various kits (all kits provided by Shanghai Yuanye Biological Co. Ltd., China). Light microscopy (Leica, Germany) was used for histopathology examination of mammary gland tissues as described [24].

### 2.8. Immunohistochemistry and Immunofluorescence Assays

The mammary gland tissues from all mothers (3 replicates each) were immediately fixed in 10% formaldehyde solutions for 24 hours before being embedded in paraffin. The immunohistochemistry (IHC) preparation was done as described previously by [25]. The mammary gland tissues were cut into 4 µm slices. The sections were permeabilized in TNB-BB (0.2% saponin, 0.1 M Tris, pH 7.5, 0.15 M NaCl/0.5% blocking agent/0.3% Triton X-100). Then, rabbit primary antibodies (1:80) of estrogen receptor (ER-*α*) were incubated with mammary gland tissue sections overnight at 4°C. After washing with PBS, the sections were incubated with the biotin-conjugated IgG secondary antibody (1:100) for 30 minutes at 37°C. The sections were incubated with the substrate for 30 minutes before being stained with DAB and counterstained with hematoxylin. A light microscope (Olympus BX-51, Tokyo, Japan) was used to evaluate the immunostaining intensity in mammary gland tissue slices from each dam. The ER-*α* protein level was measured in the alveolar cells as a percentage of positive cells relative to the total tested alveolar cells. All the antibodies were provided by Beijing Bioss Co. (China). The IHC is determined by comparing the estimation of immunoreactive cells percentage (amount score) with the estimation of staining strength (staining intensity score) as follows: the nonstained cells got 0 scores, 0.1–10% got 1 score, 10.1–50% got 2 scores, 50.1–80% got 3 scores, and 80.1–100% got 4 scores. The strength of staining is graded on a scale of 0–3, with 0 being negative, 1 being mild, 2 being moderate, and 3 being intense. When there is multifocal immunoreactivity and considerable variations in staining intensity across foci, the average of the least and most strong staining was reported. The original data were converted to the IHC by multiplying the quantity and staining intensity scores.

The immunofluorescence assay was performed as previously described by [26]. The tissue sections were treated with 4% methanol hydrogen peroxide for 20 minutes at room temperature. The tissues were incubated with antibodies of *α*-Lactalbumin (*α*-Lactalb) overnight at 4°C. Then, the sections were incubated with the secondary antibody (IgG) for 20 minutes at 37°C, washed with PBS, and dried. The experimental results were evaluated using light microscopy (Olympus IX71 inverted fluorescence microscope, Japan).

### 2.9. Real-Time Quantitative PCR Analysis

An optimized RT–PCR technique was utilized to evaluate the relative expression levels of *β*-Cas, *α*-Lactalb, prolactin receptor (PRLR), and STAT-5a RNA in the mammary gland. Approximately 50 to 100 mg of mammary glands were collected from the studied groups (CON, MET, HP, MP, LP, HW, MW, and LW) and homogenized in TRIzol (Invitrogen Co., United States) according to the manufacturer's protocol to extract RNA. The quality of RNA was judged on agarose gel electrophoresis. A260 determined the total RNA concentration, and RNA was stored at 80°C until required. Equal quantities (4 µg) of total RNA were reverse-transcribed into cDNA using a TIANScript RT Kit (Tiangen Co., China). The samples were tested in triplicate, with a negative control sample containing no reverse transcriptase (the negative control sample to determine genomic DNA). The pairs of primers used to amplify *β*-Cas, *α*-Lactalb, PRLR, and STAT-5a in addition to the GAPDH (housekeeping gene) and the reaction system details are presented in the supplementary Tables 1–3. The primers were designed by primer premier 5 software from the sequences provided by the NCBI database. The PCR system was performed as follows: after denaturing the samples at 95°C for 15 minutes, the reaction mixture was exposed to 40 cycles of denaturation at 95°C for 10 seconds, annealing at 58°C for 30 seconds, and extension at 72°C for 30 seconds. Dissociation curves were used to validate the purity of the product, and agarose gel electrophoresis was used to separate the samples. The *β*-Cas, *α*-Lactalb, PRLR, and STAT-5a expression in mammary glands relative to the GAPDH gene as an appropriate reference gene was determined using the delta-delta Ct' (Ct) equation [27].

### 2.10. Western Blotting

Western blot was performed to examine the qualitative and quantitative properties of the proteins as described by Martins-Gomes and Silva [28]. As follows: mammary gland tissues were removed from 5 dams in each group and promptly frozen in liquid nitrogen to isolate total protein. Per 20 mg of tissue, 200–400 mL of cell lysate buffer (150 mM NaCl, 50 mM Tris Base, 0.1% SDS (pH 8.0) was added. The tissues were homogenized with a hand-held electric tissue cell homogenizer (approximately 1 minute) and then centrifuged at 10625 × *g* (12,000 rpm)/10 minutes. The protein present in each cell sample was measured by the Coomassie brilliant blue method [29]. Each protein sample was resolved in 15% SDS polyacrylamide denaturing gel and then transferred to PVDF fluorine. The membranes were treated with antibodies against *β*-Cas (1:200), *α*-Lactalb (1:500), and PR (1:200). PRLR (1:100), STAT-5a (1:600), and GAPDH (1:1,000) overnight at 4°C. After that, the membrane was rinsed in TBST and incubated with a secondary antibody (Beijing Zhong Co.) in a dilution of (1:3,000) for 1 hour at room temperature while gently shaking. The correct bands were determined according to the molecular weight compared to the marker. ImageJ software was used to evaluate the grayscale values.

### 2.11. Statistical Analysis

All the data were analyzed by SPSS 20 software (Chicago, IL, USA) as the mean ± standard deviation (SD). Analysis of variance (ANOVA) was performed, depending on the normality (Shapiro–Wilk's test) [30] and/or homoscedasticity (Levene's test) of the data [31]. Followed by Duncan's post hoc test, to evaluate the significance of the differences between experimental groups. Additionally, the GraphPad Prism 8 program was used to draw graphs.

## 3. Results

### 3.1. Effect of *Leucocalocybe mongolica* Extracts on Cell Viability

**Figure 1:**
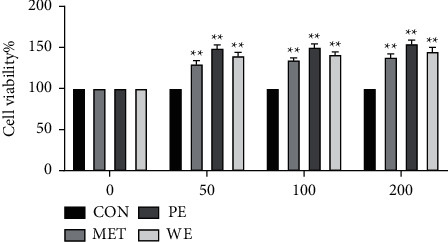
Effect of *L*. *mongolica* extracts on cell viability. MCF10 A cell lines were treated with *L*. *mongolica* petroleum ether extract (PE), water extract (WE), metoclopramide (MET), and CON control (50, 100, and 200 µg/mL) for 48 h. The cell viability was measured by CCK8. The values are represented as the mean ± SD standard deviation of three independent experiments. Compared with the CON group,  ^*∗*^*P* < 0.05 and  ^*∗*^ ^*∗*^*P* < 0.01. The normality and homoscedasticity of the data were verified using the Shapiro–Wilk and Levene's tests, respectively.

Petroleum ether and water extracts increased cell viability of MCF10 A cell line up to 30% after 48 h of incubation. Petroleum ether, water extract, and metoclopramide significantly (*P* < 0.05) increased cell viability compared with the cells were untreated cells. In addition, petroleum ether and water extract significantly (*P* < 0.05) enhanced cell viability compared with the cells were treated MET. The treated cells with petroleum ether, water extract, and metoclopramide showed a significant increase in cell viability (*P* < 0.05) compared with the untreated cells. Moreover, petroleum ether extract (50, 100, and 200 µg/mL) increased cells viability (up to 33%, 33.7%, and 35.44%, respectively (Figure 1). Also, water extracts increased cell viability (up to 29%, 32%, and 33%, respectively (Figure 1)). Thus, *L*. *mongolica* extracts were able to increase cell viability.

### 3.2. The Production of Milk and Milk Composition

**Figure 2:**
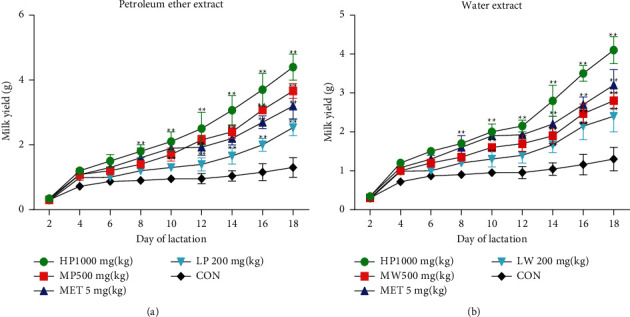
Effects of *L*. *mongolica* extracts on milk production for 18 days. The values are represented in the chart as mean ± SD standard deviation. Compared with the CON group,  ^*∗*^*P* < 0.05 and  ^*∗*^ ^*∗*^*P* < 0.01. The normality of the data within the groups was verified using the Shapiro–Wilk test, and the homoscedasticity was checked by Levene's tests.

To evaluate the effect of *L*. *mongolica* extracts on milk yield and milk composition, milk yield was calculated every day during the experiment period. The results showed significant improvement in milk production in all treatment groups compared to the CON group. With the progression of lactation, the variance between the control and treatment groups increased progressively. As shown in (Figure 2(a)), milk production in the HP and MP groups increased more than in the CON and MET groups, and the LP group possessed a higher milk yield than the CON group (*P* < 0.05). As shown in (Figure 2(b)), the milk yield in the HW group was higher than that in the MET group. In contrast, the MW and LW groups were lower than the MET group. Furthermore, total protein, fat, carbohydrate, and calcium were determined in the milk samples by ELISA kit. Milk fat is a key lipid source and provides a significant proportion of the calories and necessary and beneficial fatty acids required for newborn growth and development [32]. The results showed that the treatment groups had substantially higher total protein, total fat, and calcium contents than the control group (*P* < 0.05) (Table 1). The values of these parameters in the HP group were more significant than those in the MET group. In contrast, the total carbohydrate content in the HW and MW groups rose considerably compared with MET.

### 3.3. Growth Rate and Weight Gain of Pups

The increased weight of the pup indicates an enhancement in the nutritional composition of the dam's breastfeeding. The treatment groups demonstrated a substantial growth rate and weight increase compared with the control group. All doses of petroleum ether extract and water extract groups showed an increase in weight gain and final pup weight compared with the MET group (Table 2).

### 3.4. Serum Hormones Concentration

Prolactin plays a vital role in milk production and maintains an active gland, released through a neuroendocrine reflex to suckling stimuli [33]. We examined the effect of extracts on serum prolactin (PRL), growth hormone (GH), progesterone (Pg), and estradiol (E2) levels production by ELISA [34]. The results showed that PRL and GH levels were upregulated in the treatment groups compared with the CON group (*P* < 0.05). According to the findings in Table 3, PRL and GH hormone levels increased significantly (*P* < 0.05) in the HP and HW groups compared with the MET group. In contrast, the Pg and E2 hormones were downregulated in the treatment groups compared with the CON group (*P* < 0.05).

### 3.5. Histopathological Changes

**Figure 3:**
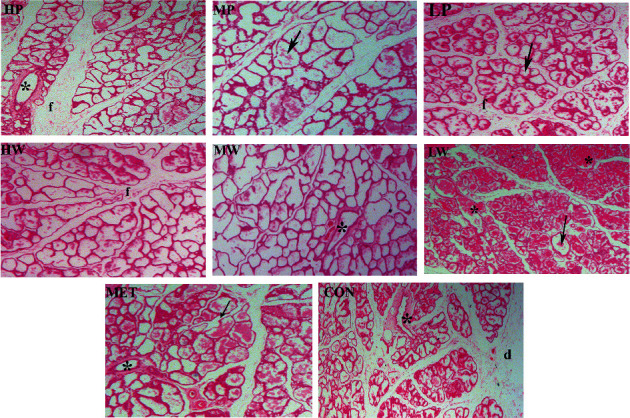
Representative images of hematoxylin and eosin-stained sections of mammary alveolar tissue from the lactating with H&E (400×). The arrows refer to the active alveoli with lobules;  ^*∗*^to the inter lobules duct; f refers to internal lobular connective tissue; and d refers to the adipose tissue.

Histopathology was performed to determine the effect of *L*. *mongolica* extracts on lobuloalveolar system development. The analysis of histological sections revealed that the dams in all extract-treated groups and MET possessed a higher score for mammary gland histological distinction than the control by increased active alveoli with lobules and the interlobular duct as well as reduced the area of adipose tissue. The higher scores were mostly related to more distended alveoli and exhibited growth in the size of lobules (Figure 3). Thus, a larger alveolar lumen in the treatment groups indicates that the mammary gland has advanced in differentiation and led to an increase in milk protein synthesis, similar to the findings of previous research [35].

### 3.6. Analysis of Immunofluorescence and Immunohistochemistry

**Figure 4:**
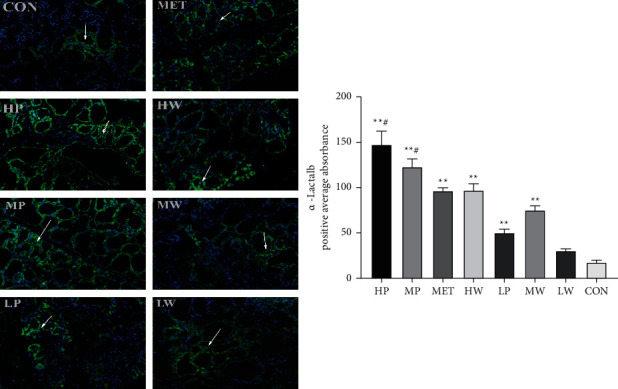
Representative immunofluorescence images of rat mammary alveoli and positive average absorbance indicating *α*-Lactalb expression. The alveolar cell's cytoplasms and apical lumens were positive for *α*-Lactalb (green), while their nuclei were negative (blue, DAPI+). The mean ± SD standard deviation is used to express the values. (Compared with the CON group,  ^*∗*^*P* < 0.05,  ^*∗*^ ^*∗*^*P* < 0.01, and  ^#^*P* < 0.05 compared with the MET group.

To understand the mechanisms that increased milk yield, milk protein composition, and synthesis of milk protein analysis, immunofluorescence and immunohistochemistry were used to detect the changes in mammary gland tissue in the treated animals compared with the CON group. The green-stained cytoplasms and nuclei mean the cells are positive for *α*-Lactalb expression, while the blue nucleus is negative. In addition, an increase in *α*-Lactalb expression mRNA was associated with an increase in *α*-Lactalb expression synthesis, as evidenced by an increased intensity of expression *α*-Lactalb expression in mammary tissues. The immunofluorescence findings showed that the positive average absorbance of *α*-Lactalb expression was upregulated in the treated groups compared with the CON group. Moreover, the positive average absorbance of *α*-Lactalb expression in the HP and MP groups was significantly higher than in the MET group (Figure 4).

**Figure 5:**
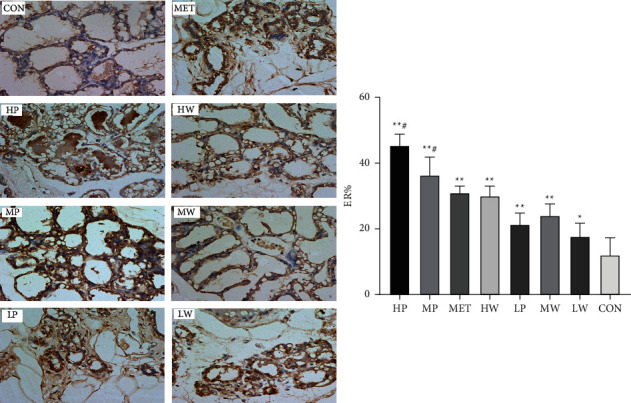
The immunohistochemistry staining (200x) and percentages of alveolar cells positive for ER-*α* in the rat mammary tissue. The bar chart expresses the mean values ± SD.  ^*∗*^*P* < 0.05, compared with the CON group, and  ^#^*P* < 0.05 compared with the MET group.

In addition, immunohistochemistry analysis showed the presence of steroid receptors positive as a brown nucleus in quantified and appearance. The percentages of alveolar cells expressing ER-*α* in the mammary glands were enhanced in all treatment groups, as shown in Figure 5. Compared to the MET group, ER expression was substantially higher in the HP, MP, and groups.

### 3.7. Effect of *Leucocalocybe mongolica* Extracts on mRNA Expression of *β*-Cas, *α*-Lactalb, PRLR, and STAT-5a

**Figure 6:**
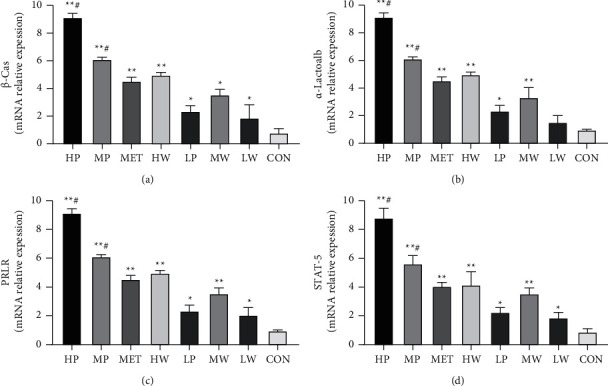
mRNA expression of *β*-Cas (a), *α*-Lactalb (b), PRLR (c), and STAT-5 (d) by real-time PCR. The bars represent the mean values ± SD. Compared with the CON group,  ^*∗*^*P* < 0.05,  ^*∗*^ ^*∗*^*P* < 0.01, and  ^#^*P* < 0.05 compared with the MET group.

The mRNA expression of *β*-Cas, *α*-Lactalb, PRLR, and STAT-5a was detected by real-time PCR to verify their expression in each group. *β*-Cas, *α*-Lactalb, PRLR, and STAT-5a mRNA expression was assessed in mammary gland samples from all dams. All treated groups remarkably increased *β*-Cas, *α*-Lactalb, PRLR, and STAT-5a transcript mRNA compared with the control group in a dose-dependent manner. Moreover, the transcript mRNA levels of *β*-Cas, *α*-Lactalb, PRLR, and STAT-5a in the HP, MP, and HW groups were higher than those in the MET group, while the expression levels of both genes in the LP, MW, and LW groups were lower (Figure 6).

### 3.8. Effect of *Leucocalocybe mongolica* Extracts on mRNA Expression of *β*-Cas, *α*-Lactalb, PRLR, and STAT-5a Proteins

**Figure 7:**
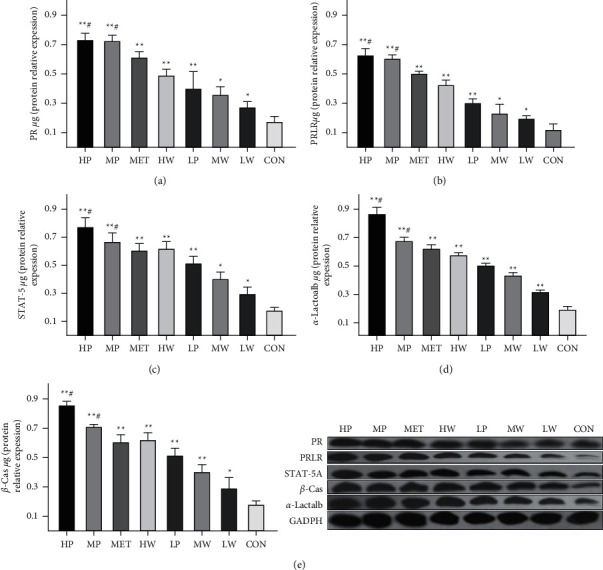
The expression levels of PR (a), PRLR (b), STAT-5a (c), *α*-Lactalb (d), and *β*-Cas (e) proteins in the mammary tissues and typical images of western blots. The data are expressed per µg of tissue, and the bars indicate the mean values ± SD. (Compared with the CON group,  ^*∗*^*P* < 0.05,  ^*∗*^ ^*∗*^*P* < 0.01, and  ^#^*P* < 0.05 compared with the MET group.

The expression levels of *β*-Cas, *α*-Lactalb, PRLR, and STAT-5a proteins were detected by western blot. The western blot results confirmed the findings by rtPCR that the expression levels of *β*-Cas, *α*-Lactalb, PRLR, and STAT-5a in the extract-treated groups were higher than those in the control group in a dose-dependent manner. Moreover, the expression of *β*-Cas, *α*-Lactalb, PRLR, and STAT-5a proteins in the HP, HW, and MP groups was higher than that in the MET group (Figure 7). This result is also similar to the immunohistochemistry analysis results seen in the alveolar lumen (Figure 5). Overall, our results indicate that crude extracts could improve milk production and protein synthesis by increasing milk protein expression levels.

## 4. Discussion

Breast milk is considered the optimal nutrition for infants in the first six months. It mainly consists of water, milk proteins, fatty globules, lactose, calcium, and other compounds [36]. The composition of mother's milk is significantly affected by environmental variables and mother's nutrition [37]. However, many postpartum mothers have hypogalactia after motherhood due to mental stress, worry, and diseases [38]. Thus, mothers need breastfeeding support to increase their milk production. Metoclopramide is a popular galactagogue medicine that increases prolactin levels and enhances milk production [39]. However, there is a warning label on the medicine's box about late dyskinesia when the medication is used for more than three months [38].

Some natural medicines can augment or trigger milk production [40]. Our results support the ethnopharmacological application of *L*. *mongolica* extracts for galactagogue functions in nursing mothers. The results showed that a high dose of petroleum ether extract and water extract of *L*. *mongolica* significantly improved milk production and weight gain compared to the MET group. *L*. *mongolica* contains nutritional components such as vitamins, proteins, fiber, fatty acids, and many minerals, in addition to the main bioactive components, such as polysaccharides, ergosterol, lectins, and laccase, which are found in large quantities [19, 21, 22, 41, 42]. The higher dosage of petroleum ether and water extract of *L*. *mongolica* increased the milk content of total fat, total protein, total carbohydrate, and calcium compared to the MET group (Table 1). Previous studies found that appropriate milk composition depends on the optimal biochemical and structural differentiation of alveolar cells [43]. However, the results revealed that the doses of petroleum ether extract showed higher milk proteins, fats, carbohydrates, and calcium content than the equal dose of water extract.

**Figure 8:**
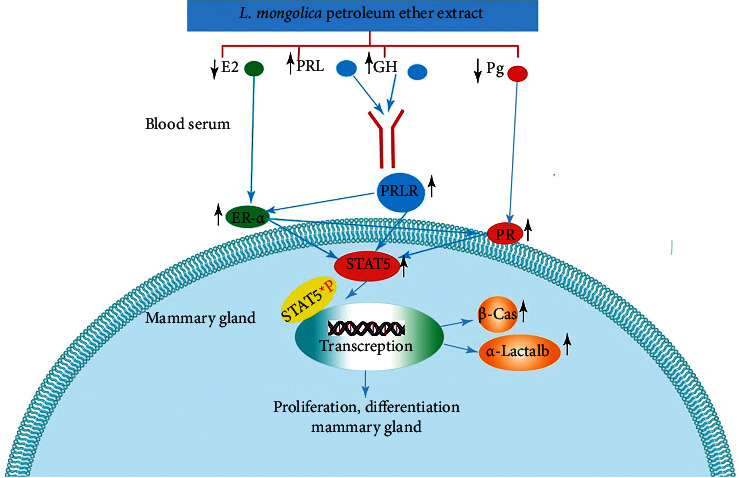
The mechanism by which *L*. *mongolica* improves mammary gland proliferation and increases milk protein expression.

Thus, several hormones play a significant role in the regulation of milk synthesizing processes, and the expression of receptors for certain hormones in mammary tissue indicates their direct involvement. Thus, the results revealed that petroleum ether and water extract remarkably increased lactogenic hormones, prolactin, and growth hormone. The results in this study support the findings of earlier research, which indicated that the increase in prolactin levels was associated with the maintenance of lactation and enhanced breast milk production [44, 45]. Serum prolactin level during breastfeeding was associated with the development of alveoli of mammary lobules [46]. Similarly, the administration of prolactin increased milk secretion in rabbits, especially in the early lactation period [47, 48]. In addition, GH is required for mammary gland growth and milk production [49]. GH and PRL are necessary for the proliferation and differentiation of mammary gland epithelial cells and the expression of milk proteins [50]. A previous study showed that increased hormone levels of GH and PRL during lactating can improve alveoli distension in the mammary gland, enhancing its capacity to accommodate increased milk volume [34]. Progesterone and estrogen are responsible for mammary epithelial tissue proliferation and lobuloalveolar development via conserving the expression of STAT-5A and activating the development of milk canals (Figure 8) [51]. In our study, petroleum ether and water extract downregulated progesterone and estradiol hormone. The progesterone and estradiol hormone levels in the treated rats with petroleum ether extracts were lower than similar water extract concentrations, while lactogenic hormones, prolactin, and growth hormone levels were higher in petroleum ether extracts in all the concentrations. As described above, serum prolactin levels during breastfeeding are associated with the development of alveoli of mammary lobules [46]. The histological result showed that petroleum ether and water extract exhibited growth in the size of lobules (Figure 3). Thus, a larger alveolar lumen in the treatment groups indicates that the mammary gland has advanced in differentiation, leading to an increase in milk protein synthesis, similar to the findings of research [35].

Earlier studies found that the water extract of *L*. *mongolica* contains lectins, laccase, and polysaccharides [21, 42, 52]. The polysaccharides from *L*. *mongolica* possess antioxidant immune and antitumor properties [18, 42, 53]. Lectins from *L*. *mongolica* possessed potent hypotensive and vasorelaxant activities [54]. On the other hand, the petroleum ether extract of *L*. *mongolica* contains steroids [22]. Some steroids in the petroleum ether extract promote lactation and may improve the use of plants as galactogogues by stimulating the production of lactation hormones [40]. However, ergosterol and laccase from this mushroom inhibited the growth of MCF7 cells in breast cancer [21, 22]. More significantly, ergosterol and several metabolic intermediates are key metabolites with high economic importance. Ergosterol is used in the pharmaceutical sector as a precursor for vitamin D2 and steroid hormone biosynthesis [55]. For instance, ergosterol can be used to generate cortisone and progesterone [56]. Therefore, *L*. *mongolica* has high antioxidant properties [57]. All these bioactive compounds enhance the nutritional value of *L*. *mongolica*. The higher nutritional value is known to promote milk output [58].

The development and differentiation of the mammary gland is a key event involved in increasing milk yield and milk protein synthesis. However, PR is a protein dimerization transcription factor that can bind the target genes as dimers. PR is limited to the luminal cell line without expression in mammalian myoepithelial compartments [59]. PR and PRL can regulate the activation of the transcription of these genes [60] in the mammary gland [13]. According to the immunohistochemistry results, the expression of ER was increased in all treatment groups. ER is frequently coexpressed in the mammary gland, which indicates a certain percentage of epithelial cells expressing steroid receptors [61].

Furthermore, the transcript mRNA levels of *β*-Cas, *α*-Lactalb, PR, PRLR, and STAT-5A were increased in the HP, MP, HW MW, LP, and LW groups compared to the control group. The HP, MP, and LP possess higher expression levels for all the studied genes than HW, MW, and LW, respectively. *β*-casein is a transcriptional target of STAT-5a downstream of prolactin receptor signaling [62]. Our findings indicate that the extracts may potentially upregulate the *β*-Cas, and *α*- Lactalb, transcription. These findings suggested that *L*. *mongolica* extracts may increase milk protein synthesis. The expression of the *α*-Lactalb gene is required for lactose synthesis, and *α*-Lactalb integrates into the lactose synthesizing system to function correctly. Due to the relationship between prolactin and the systemic synthesis of lactose, during pregnancy and lactation, *α*-Lactalb expression appears exclusively in the mammary gland [63].

As discussed above, Pg and E2 synergize in the stimulation of mammary cell proliferation and differentiation. Previous studies found a strong positive correlation between estradiol, progesterone, and PRLR levels and mammary gland differentiation via regulating various signaling pathways (Figure 8). These steroids antagonize the effect of PRL on the synthesis of milk proteins during pregnancy, whereas they have very little impact during lactation. Estradiol activates estrogen receptor alpha (ER-*α*), leading to rising progesterone receptor expression and resulting in progesterone activation. In the same way, PRL and the nuclear PRLR levels can enhance the expression of ER-*α* and progesterone receptors (PRs) [64, 65]. Prolactin and progesterone are essential regulators of STAT proteins. Moreover, both prolactin and progesterone enrich prolactin-mediated stimulation of STAT protein activation in part via the Janus kinase (JAK2) and mitogen-activated protein kinase (MAPK) pathways by multiplying convergent signaling pathways and inserting phosphorylation to STAT [66]. The phosphorylation of STAT-5a stimulates alveolar lineage development. STAT5a is a master regulatory factor of alveologenesis, milk protein genes, and Elf5 expression [67]. STAT-5a has a notable impact on establishing the luminal progenitor cell division in the mammary gland [68, 69]. All of the above evidence confirms the efficacy and mechanism of *L*. *mongolica* in improving the growth of the mammary gland and protein synthesis.

## 5. Conclusion

The present study clarified that petroleum ether and water extracts of *L*. *mongolica* improve milk yield and mammary gland differentiation by upregulating *β*-Cas and *α*-Lactalb and steroid receptor expression. This investigation showed that water and petroleum ether extracts increased milk yield and milk content of calcium, total fat, carbohydrate, and protein compared to control. Also, serum PRL and GH levels were significantly raised in all treated groups compared with the control group, while Pg and E2 levels were decreased. Similarly, the expression levels of *β*-Cas, *α*-Lactalb, PRLR, PR, and STAT-5a in the treated groups were higher than control and MET in a dose-dependent manner. This study provides scientific evidence for the biological functions of *L*. *mongolica* as well as the potential of medicinal and nutritional properties and how they might be used to increase milk production and quality. Overall, the results illustrated that petroleum ether extracts were better than water extracts in improving milk yield and mammary gland differentiation.

## Figures and Tables

**Table 1:** Effect of the extracts on milk content of total fat, total protein, total carbohydrate, and calcium.

Treatment	Total fat (µg/mL)	Total protein (µg/mL)	Total carbohydrate (ng/mL)	Calcium (mg/mL)
CON	17.7 ± 1.8	27.7 ± 2.2	263.3 ± 1.3	0.6 ± 0.08
MET	22.2 ^*∗*^ ^*∗*^ ± 0.8	48.5 ^*∗*^ ^*∗*^ ± 1.5	294.72 ± 2.5	1.03 ^*∗*^ ^*∗*^ ± 0.1
HP	34.8 ^*∗*^ ^*∗*^ ± 2.2	54.1 ^*∗*^ ^*∗*^ ^#^ ± 1.9	337.71 ^*∗*^ ^*∗*^ ± 1.71	1.12 ^*∗*^ ^*∗*^ ^#^ ± 0.08
MP	21.2 ^*∗*^ ^*∗*^ ± 1.2	34.8 ^*∗*^ ^*∗*^ ± 0.8	312.31 ^*∗*^ ^*∗*^ ± 2.4	0.95 ^*∗*^ ^*∗*^ ± 0.05
LP	21.1 ^*∗*^ ^*∗*^ ± 0.8	36.1 ^*∗*^ ^*∗*^ ± 1.1	268.3 ± 1.8	0.86 ^*∗*^ ^*∗*^ ± 0.03
HW	28.8 ^*∗*^ ^*∗*^ ± 0.7	50.1 ^*∗*^ ^*∗*^ ± 0.6	426.68 ^*∗*^ ^*∗*^ ^#^ ± 2.1	1.01 ^*∗*^ ^*∗*^ ± 0.06
MW	21.9 ^*∗*^ ^*∗*^ ± 1.5	45.9 ^*∗*^ ^*∗*^ ± 1.7	356.30 ^*∗*^ ^*∗*^ ^#^ ± 2.8	0.9 ^*∗*^ ^*∗*^ ± 0.17
LW	18.7 ± 1.7	38.7 ^*∗*^ ^*∗*^ ± 1.1	346.04 ^*∗*^ ^*∗*^ ± 3.3	0.88 ^*∗*^ ^*∗*^ ± 0.03

The mean ± SD standard deviation is used to express the values. (Compared with the CON group,  ^*∗*^*P* < 0.05,  ^*∗*^ ^*∗*^*P* < .01 and  ^#^*P* < 0.05 compared with the MET group).

**Table 2:** Differences between the mean initial and final weights of pups.

Group	Initial weight (g)	Final weight (g)	Weight gain (g/pup)
CON	9.20 ± 0.48	49.57 ± 1.89	2.24 ± 0.11
MET	9.07 ± 0.76	52.64 ± 2.00	2.42 ± 0.08
HP	9.07 ± 0.63	62.92 ^*∗*^ ^*∗*^ ^#^ ± 3.7	3.09 ^*∗*^ ^*∗*^ ^#^ ± 0.18
MP	8.96 ± 0.42	61.95 ^*∗*^ ^*∗*^ ^#^ ± 1.60	2.94 ^*∗*^ ^*∗*^ ^#^ ± 0.09
LP	8.89 ± 0.74	60.29 ^*∗*^ ^*∗*^ ^#^ ± 1.86	2.86 ^*∗*^ ^*∗*^ ^#^ ± 0.13
HW	8.89 ± 0.51	61.21 ^*∗*^ ^*∗*^ ^#^ ± 1.91	2.91 ^*∗*^ ^*∗*^ ^#^ ± 0.11
MW	8.16 ± 0.41	60.83 ^*∗*^ ^*∗*^ ^#^ ± 3.47	2.81 ^*∗*^ ^*∗*^ ^#^ ± 0.19
LW	8.60 ± 0.23	58.66 ^*∗*^ ± 3.24	2.78 ^*∗*^ ^*∗*^ ^#^ ± 0.19

The mean ± SD standard deviation is used to express the values based on *n* = 6. Compared with the CON group,  ^*∗*^*P* < 0.05,  ^*∗*^ ^*∗*^*P* < 0.01, and  ^#^*P* < 0.05 compared with the MET group.

**Table 3:** The various hormone concentrations in serum of dams.

Treatment	PRL (ng/mL)	GH (µg/mL)	Pg (ng/mL)	E2 (ng/mL)
CON	42.13 ± 3.30	2.50 ± 0.34	18.57 ± 1.4	13.17 ± 2.93
MET	65.20 ^*∗*^ ^*∗*^ ± 1.29	4.09 ^*∗*^ ^*∗*^ ± 0.10	16.23 ^*∗*^ ± 1.37	9.838 ^*∗*^ ± 1.26
HP	85.62 ^*∗*^ ^*∗*^ ^#^ ± 0.88	4.46 ^*∗*^ ^*∗*^ ^#^ ± 0.17	12.43 ^*∗*^ ± 1.12	8.84 ^*∗*^ ± 1.27
MP	68.74 ^*∗*^ ^*∗*^ ± 1.33	3.81 ^*∗*^ ^*∗*^ ± 0.24	14.63 ^*∗*^ ± 0.65	9.07 ^*∗*^ ± 0.89
LP	64.39 ^*∗*^ ^*∗*^ ± 5.60	3.38 ^*∗*^ ^*∗*^ ± 0.12	18.4 ± 1.27	11.1 ± 0.56
HW	71.68 ^*∗*^ ^*∗*^ ^#^ ± 3.97	4.40 ^*∗*^ ^*∗*^ ^#^ ± 0.10	14.93 ^*∗*^ ± 0.61	8.87 ^*∗*^ ± 0.81
MW	50.56 ^*∗*^ ± 2.09	3.45 ^*∗*^ ± 0.11	15.43 ± 0.78	10.43 ± 0.602
LW	47.72 ± 1.09	2.94 ^*∗*^ ± 0.41	17.43 ± 1.10	12.5 ± 1.3

The data obtained after 20-day treatment. The mean ± SD standard deviation is used to express the values. Compared with the CON group,  ^*∗*^*P* < 0.05,  ^*∗*^ ^*∗*^*P* < 0.01, and  ^#^*P* < 0.05 compared with the MET group.

## Data Availability

The data used to support the findings of this study are available from the corresponding author upon request.

## Abbreviations

HP: Petroleum ether extract at a high dose
MP: Petroleum ether extract at a medium dose
LP: Petroleum ether extract at a low dose
HW: Water extract for *L*. *mongolica* at a high dose
MW: Water extract for *L*. *mongolica* at a medium dose
LW: Water extract for *L*. *mongolica* at a low dose
CON: Control group
MET: metoclopramide
PRL: Prolactin
GH: Growth hormone
E2: Estradiol
Pg: Progestin
*α*-Lactalb: *α*-Lactalbumin
PR: Progesterone receptor
ER: Estrogen receptor
*β*-Cas: *β*-Casein.

## Supplementary Materials

Supplementary Materials The following supporting information can be downloaded. Table 1: The primer pairs utilized for amplification of *β*-Cas and *α*-Lactalb in the GAPDH. Table 2: The PCR mixture. Table 3: Rt-PCR system.
