# Supplementary material for: Medicinal Mushroom Leucocalocybe mongolica Imai Extracts Improve Mammary Gland Differentiation in Lactating Rats via Regulating Protein Expression
Source: Evid Based Complement Alternat Med. 2022 Jun 18;2022:5762847. doi: 10.1155/2022/5762847 (PMC9233605; doi:10.1155/2022/5762847)
Supplement: Supplementary Materials — The following supporting information can be downloaded. Table 1: The primer pairs utilized for amplification of β-Cas and α-Lactalb in the GAPDH. Table 2: The PCR mixture. Table 3: Rt-PCR system. [file 5762847.f1.docx]

Medicinal mushroom *Leucocalocybe mongolica* Imai Extracts Improve Mammary Gland Differentiation in Lactating Rats via regulating Protein Expression

Asmaa Hussein Zaki^a,c^, Bao Haiying ^*a,b^ and Li Zhijun^a, b^

Supplementary data

Table: 1 shows the primer pairs utilized for amplification of β-Cas, and α-Lactalb in the GAPDH

| **Primer name** | Sequence | gene length（bp） | Annealing Tm |
| --- | --- | --- | --- |
| beta casein-F | ACAGGTTTGCAGGACTCGAC | 141 | 58 |
| beta casein-R | AACTCCTGAGAAGCGCTGTG |  | 58 |
| alpha lactalbumin-F | GCGCAAGTGTTACGAAGTGG | 146 | 58 |
| alpha lactalbumin-R | GGTTGGCAGCTCTCATGTCT |  | 58 |
| GAPDH-F | CAGGGCTGCCTTCTCTTGTG | 186 | 60 |
| GAPDH-R | TCTCGCTCCTGGAAGATGGT |  | 60 |

Table 2: shows the PCR mixture

| Reagent | 20ul reaction system |
| --- | --- |
| 2*SuperReal PreMix Plus | 10μl |
| Forword primer （10μM） | 0.6μl |
| Reverse primer（10μM） | 0.6μl |
| cDNA | 100ng |
| 50*ROX Reference Dye△ | 0.4μl |
| RNase-Free ddH_2_O | Up to 20μl |

Table 3: Rt-PCR system

| cycles | Temprature | Time |
| --- | --- | --- |
| 1× | 95℃ | 15min |
| 40× | 95℃ | 10s |
|  | 58℃ | 30s |
|  | 72℃ | 30s |
